# Tranexamic acid for reducing blood loss in bipolar transurethral resection of the prostate: a systematic review of literature

**DOI:** 10.31744/einstein_journal/2024RW0734

**Published:** 2024-11-19

**Authors:** Jonathan Doyun Cha, Gabriel Franco de Camargo Galindo, Caroline Vidalli Denser, Carlos Henrique Alves da Silva, Arie Carneiro

**Affiliations:** 1 Hospital Israelita Albert Einstein São Paulo SP Brazil Hospital Israelita Albert Einstein, São Paulo, SP, Brazil.; 2 Hospital Israelita Albert Einstein Hospital Municipal da Vila Santa Catarina Dr. Gilson de Cássia Marques de Carvalho São Paulo SP Brazil Hospital Municipal da Vila Santa Catarina Dr. Gilson de Cássia Marques de Carvalho; Hospital Israelita Albert Einstein, São Paulo, SP, Brazil.; 3 Hospital Israelita Albert Einstein Faculdade Israelita de Ciências da Saúde Albert Einstein São Paulo SP Brazil Faculdade Israelita de Ciências da Saúde Albert Einstein, Hospital Israelita Albert Einstein, São Paulo, SP, Brazil.

**Keywords:** Transurethral resection of prostate, Prostatic hyperplasia, Hemorrhage, Tranexamic acid

## Abstract

**Objective:**

To study the efficacy of tranexamic acid at reducing bleeding during bipolar prostate resection surgery (B-TURP) in patients with benign prostatic hyperplasia.

**Methods:**

We searched registers with MESH terms "prostate hyperplasia," "prostate surgery," and "tranexamic acid." Studies available in full and online, published from 2013 to 2023, in Portuguese, English, Spanish, and French were included; review articles were excluded. Information sources: Portal Regional da *Biblioteca Virtual em Saúde* and PubMed Central. The Cochrane RoB2 tool was used to analyze risk of bias in randomized clinical trials.

**Results:**

Two randomized clinical trials involving 256 patients were included. Both groups had minimal risk of bias. Both studies showed a positive effect of tranexamic acid on blood loss parameters. Only one study in the tranexamic acid group had a lower transfusion rate, and another had a lower irrigation fluid volume and operation time in the tranexamic acid group. A meta-analysis was not performed because of the limited number of eligible studies.

**Conclusion:**

For patients undergoing B-TURP for benign prostatic hyperplasia symptoms, the use of tranexamic acid reduced blood loss. However, a limited number of patients were studied, and the available randomized clinical trials presented conflicting conclusions. Therefore, further studies are needed to explore this aspect in detail.

**Prospero database registration:**

(www.crd.york.ac.uk/prospero) under registration ID CRD42023416383.

## INTRODUCTION

Benign prostatic hyperplasia (BPH) refers to non-malignant growth of the prostate, which is commonly observed in aging men.^(1)^ It is histologically diagnosed in 50% of men aged 60 years and up to 90% of men aged 90 years.^(2)^ Patients tend to present lower urinary tract symptoms, which negatively impact quality of life and represent a cost of billions of dollars per year to the health system.^(3)^ In most cases, treatment includes observation and pharmacotherapy, but may require surgery under special conditions. Currently, the standard surgical treatment for urologists is transurethral resection of the prostate (TURP).^(4)^

Various classical surgical techniques have been developed to meet special needs and reduce complications.^(5)^ In transurethral resection of the prostate using bipolar energy (B-TURP), saline can be used as an irrigation fluid, which decreases the occurrence of water intoxication, does not stimulate the obturator nerve, and does not interfere with cardiac devices.^(6,7)^

Other available techniques include endoscopic enucleation of the prostate (for example, using Plasmakinetics or Holmium laser - HoLEP), vaporization of the prostate (using Green Light laser or thulium laser), UroLift, Aquablation, Rezum, Prostate artery embolization, and laparoscopic/robot-assisted simple prostatectomy, which can be performed depending on the patient, surgeon's expertise, surgery center availability, and other factors.^(4,8,9)^ Recent studies have assessed the feasibility and safety of surgical BPH treatment in outpatient settings, and showed that TURP, HoLEP, and Green Light Laser Vaporization can achieve promising results.^(10)^

The most common complication of TURP is hemorrhage, which requires blood transfusion in up to 7.1% of cases.^(11)^ The hyperplasic prostate tissue has rich vascularization, and urine and urothelium release high concentrations of plasminogen activators, which stimulate the fibrinolytic system.^(12)^ Considering that fibrinolysis is a determinant of blood loss related to surgery, the use of antifibrinolytics, such as tranexamic acid (TXA), has been explored.^(13-15)^

The association between the advantages of B-TURP and the probable anti-hemorrhagic effect of TXA could be beneficial for patients and the health system as a whole, considering that it could avoid a chain of complications initiated by hemorrhage, with potential reduction in hospital costs and hospitalization duration. Meta-analyses on the use of TXA during prostate surgeries have analyzed various surgical techniques without considering their particularities.^(13-16)^ The present study narrows these investigations by analyzing only studies that performed B-TURP to better guide clinical practice.

### Research question

Does tranexamic acid reduce intra- and post-operative blood loss in patients undergoing B-TURP for benign prostatic hyperplasia?

## OBJECTIVE

To analyze the evidence in literature regarding the efficacy of tranexamic acid in reducing bleeding in patients undergoing bipolar prostate resection surgery for benign prostatic hyperplasia.

## METHODS

### Study design

The present study was a systematic literature review performed according to the Preferred Reporting Items for Systematic Reviews and Meta-Analysis (PRISMA).^(17)^

### Data sources and search strategy

The databases *Portal Regional da Biblioteca Virtual em Saúde* (BVS) and PubMed Central (PMC) of the *National Center of Biotechnology Information* (NCBI-NIH) were searched on March 15, 2023. In both databases, the descriptors (DECS/MESH) in English "prostate hyperplasia" and "prostate surgery" were used, separated by the Boolean operator "OR," and "tranexamic acid" separated by the Boolean operator "AND." The records were analyzed by one researcher and revised by a second. Any disagreements were judged by a third (senior) researcher.

### Eligibility criteria

After excluding duplicates, the studies were independently screened for eligibility by two investigators. Potentially eligible studies were screened by title and abstract; if adequate, the full text was analyzed. Studies were included if they met the following criteria. Inclusion: randomized clinical trials (RCT), intervention with Control Group, intervention without Control Group, case report, case series, and cohort studies; available in full and online; published from January 1, 2013 to March 15^t^, 2023; and published in the languages Portuguese (Brazil), English, Spanish, and French. The exclusion criteria were as follows: articles that did not specify the surgical technique used, narrative reviews, systematic reviews, meta-analyses, posters, and abstracts.

### Analysis of the study quality

The Cochrane RoB2 tool was used for analyzing the risk of bias in randomized controlled trials.^(18)^ Risk of bias 2 (RoB 2) is structured into a fixed set of domains of bias, focusing on distinct aspects of trial design, conduct, and reporting. Within each domain, a series of questions (‘signaling questions’) aim to elicit information about the trial features relevant to the risk of bias. A proposed judgement regarding the risk of bias arising from each domain is generated by an algorithm based on answers to the signaling questions. The analysis is presented in a summary table as "high risk," "low risk," or "some concerns."^(18)^ The assessment was independently performed by two reviewers. Any disagreements were resolved by consulting the senior author.

### Data processing and result generation

The StART application (http://lapes.dc.ufscar.br/) was used for the protocol elaboration, study selection, and data extraction. The data were extracted by a single researcher and revised by a second researcher. Any disagreements were solved by a third (senior) researcher. The results were generated in the form of a summary table, considering the chronological order of publications. The primary information extracted included the number of participants, age (mean, years), prostate size (g), preoperative hemoglobin level (g/dL), and TXA administration protocol. Data using another unit of measure were converted to the pre-specified unit. In case of missing data, this information is stressed in the additional descriptive analysis. The primary outcome investigated was a drop in hemoglobin levels (g/dL), and the secondary outcomes were days of hospitalization, intraoperative blood loss (mL), surgery duration (min), blood transfusion (number of events), and volume of irrigation fluid used (L).

## RESULTS

**Figure 1:**
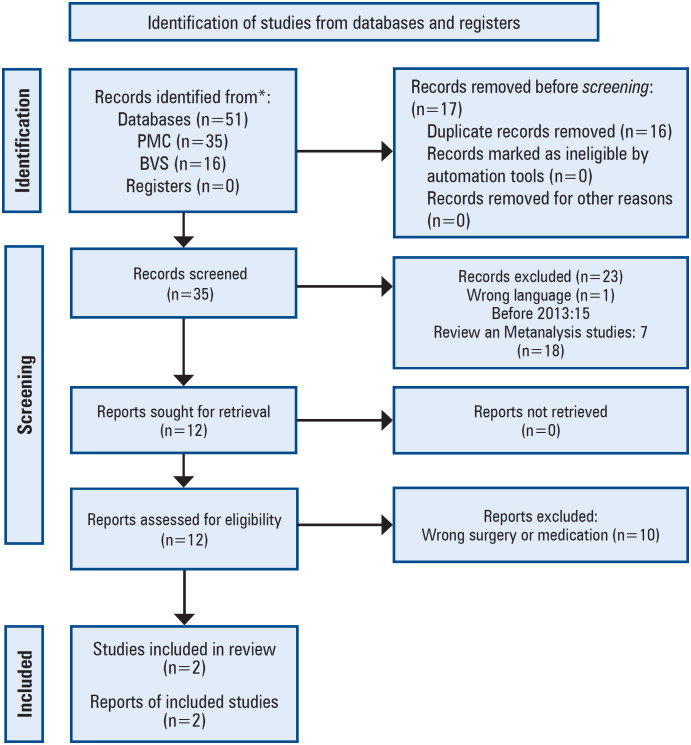
PRISMA 2020 flow diagram

A summary of the screening and selection processes is shown in figure 1. We identified 51 registers for the first query. After removing duplicates, 35 records were screened using our eligibility criteria and the full texts of 12 potentially eligible articles were retrieved for further evaluation. Finally, two randomized controlled trials involving 256 patients were included.

**Figure 2:**
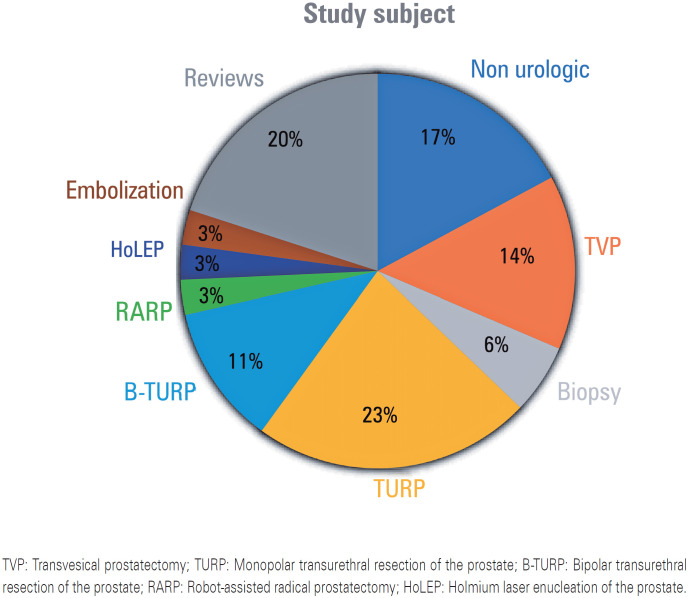
Subject of the retrieved studies TVP: Transvesical prostatectomy; TURP: Monopolar transurethral resection of the prostate; B-TURP: Bipolar transurethral resection of the prostate; RARP: Robot-assisted radical prostatectomy; HoLEP: Holmium laser enucleation of the prostate.

**Figure 3:**
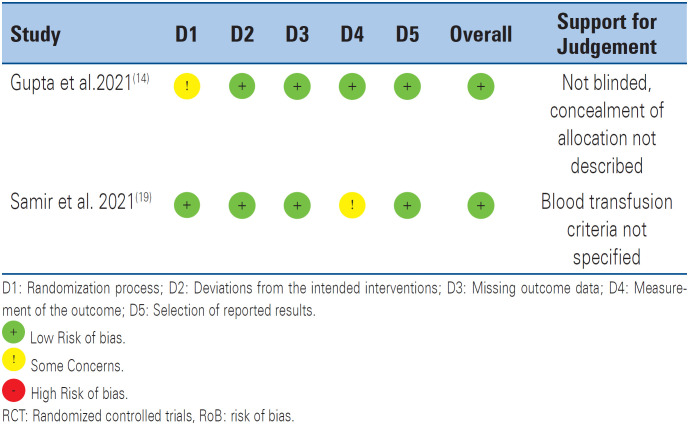
Risk of bias of included randomized clinical trials using Cochrane RoB 2

In general, the retrieved studies, including the two selected ones, reported many surgery types. Figure 2 shows the subject distribution of the 35 articles retrieved after the search (duplicate articles were excluded from this analysis). Figure 3 shows the ROB analysis performed using the RoB2 tool.

The baseline patient characteristics, TXA protocols, and outcomes of the selected studies are presented on table 1. Both trials used a 26-F bipolar resectoscope (Karl Storz®; Karl Storz SE &Co. KG, Tuttlingen, Germany), with a continuous flow and U-shaped cutting loop; the generator was adjusted to 120W for coagulation and 200W for cutting. Normal saline was used as an irrigant.

**Table 1:** Baseline characteristics of the included studies

Authors	Type	Country	Patients	Trial group	Protocol	n	Age (Years)	Prostate size (g)	Preop Hb (g/dL)	Hospital stay (Days)	Administration	Outcomes analyzed
Gupta et al., 2021^(14)^	RCT	India	Prostatic enlargement, LUTS undergoing B-TURP	Intervention	IV TXA 500 mg and 500 mg in each irrigation fluid bottle of 3 L (max 2 g)	35	68.2	56.87	13.34	3.114	Preoperative and topical	Blood loss, ΔHb, operative time, blood transfusion, hospital stay
				Control	No treatment	35	66.5	51.2	13.56	3.08	-	
Samir et al., 2021^(19)^	RCT	Egypt	Patients with prostate size 80-130g undergoing B-TURP	Intervention	IV TXA 50 mg/kgpreop, 5 mg/Kg/h maintenance dose until resection completed	95	64.66	108.32	13.12	2.33	Preoperative and intraoperative	ΔHb, operative time, blood transfusion, hospital stay
				Control	Equal volume of NaCl 0.9% infusion	91	65.75	107.09	12.59	2.37		

Hb: Hemoglobin; RCT: randomized controlled trial; LUTS: lower urinary tract symptoms; IV: intravenous; TXA: tranexamic acid; B-TURP: Bipolar transurethral resection of the prostate.

Gupta et al. in 2021^(14)^ found a significant decrease in blood loss (in mL range) during surgery when using TXA (TXA Group, 174.6±125.38 and Control Group, 232.47±116.8; p=0.04), and a significant decrease in transfusion rates. This difference was particularly significant in patients with larger prostate sizes (prostate size >60g, p=0.019). However, they did not observe any changes in the volume of irrigation fluid used, operating time, or length of hospitalization. No significant adverse effects were observed.

In 2021, Samir et al.^(19)^ studied high doses of TXA during large prostate surgeries (80-130g). In this study, the blood loss in mL was not measured, whereas ΔHb was significantly higher in the Control Group (p=0.001). They also found a significant difference in the irrigation fluid volume used (higher in the Control Group, p=0.001), mean operative time (higher in the Control Group, p=0.001), and prostate size (the TXA Group had significantly larger prostates, p=0.038). There was no difference in the transfusion rate and hospital stay. No significant adverse events were reported.

## DISCUSSION

The prostate is a abundanty irrigated organ, with large venous sinuses, and contains fibrinolytic enzymes that reach blood circulation during surgery and promote bleeding. Therefore, antifibrinolytics may play a special role in this regard.^(12)^

In recent years, TXA has proven to be efficient in reducing bleeding and the need for transfusion in many surgical fields.^(20,21)^ The risk of post-operative convulsions during cardiac procedures using high doses in older patients has been well documented.^(22)^Nevertheless, in urological procedures, including prostate surgery, TXA seems to be safe.^(21)^ The route of administration and dose of TXA varies among studies, and no standardized protocol exists for urological procedures in older patients.

The topical route of TXA administration might play a role in avoiding thromboembolic adverse events because of the lower dose absorbed. Tawfic et al. compared two groups of 25 patients each, one receiving TXA in the irrigation fluid and post operative bladder wash, and the other receiving a placebo (distilled water). The TXA Group showed significantly lower intraoperative and postoperative blood loss. However, no thromboembolic events occurred in either group.^(23)^ Gupta et al.^(14)^ associated irrigation with IV TXA but did not observe robust results. None of the included RCTs reported thromboembolic events.

The need for blood transfusion is a sign of major blood loss and contributes to surgical morbidity, mortality, and a economic impact on the healthcare system. The studies included in this review reported different results regarding this issue. Gupta et al.^(14)^ reported that TXA dramatically reduced the transfusion rate, and stressed that rigid parameters were followed when prescribing transfusion (blood loss >450mL or post-operative Hb <10mg/dL). Samir et al.^(19)^ reported that the blood transfusion rate did not differ between the groups. Notably, the prostate size in the TXA Group was significantly larger than that in the Control Group and could have interfered with blood loss, although other bleeding parameters did not show this; further, we do not know the exact threshold for transfusion in this case. A recent study on TXA use in transurethral prostate surgery concluded that transfusion rates were similar between groups (p=0.27).^(16)^

Perioperative blood loss can be measured in many ways, including mL/g of prostate tissue,^(24)^ Hemoglobin at 4 hours and 24 hours after surgery, and ml of blood dissolved in irrigation fluid. Gupta et al.^(14)^ calculated blood loss during the surgery using a predetermined formula that considered the volume of blood present in the irrigation fluid. They observed a statistically significant decrease in the TXA Group, consistent with the results of previous studies on TURP.^(13,16)^ However, Samir et al.^(19)^ did not use this method.

Both studies measured Hb loss at 24 hours after surgery. Gupta et al.,^(14)^ did not find a statistically significant difference, whereas Samir et al.,^(19)^ found a significant reduction in blood loss in the TXA Group (p=0.001). Previous studies on TURP showed no difference between groups in terms of Hb change after 24 hours.^(25)^

The operative time was the same for the groups in the study by Gupta et al.,^(14)^ while in the study by Samir et al.,^(19)^ significantly longer surgery durations were observed in in the Control Group (p=0.001), which they explained based on better hemostasis and improved vision in the TXA Group. Other studies have confirmed the latter result, achieving shorter surgeries in the TXA Groups.^(15,24)^

Finally, the volume of irrigant fluid used showed no statistically significant difference in the study by Gupta et al.^(14)^ (p=0.703) and was significantly lower in the TXA Group in the study by Samir et al.,^(19)^ consistent with the duration of the procedure reported in both studies. The majority of other trials addressing TURP found a reduction in the fluid volume used in the TXA Groups,^(15,25)^ while one trial found no differences between groups in this regard.^(26)^

Both studies showed no difference in hospital stays between the TXA and Control Groups. This is in accordance with the majority of previous trials on TURP.^(13,24,27)^ There was no evidence of gross hematuria or clot retention, which could prolong the catheterization time and length of hospitalization. They also did not report major adverse effects such as deep venous thrombosis, seizures, or myocardial infarction, despite the theoretical risk of these events when using high doses of TXA in older patients. To tackle this question, a recent metanalysis concluded that lysine analogues are effective at reducing blood loss during and after surgery and appear to be free of serious adverse effects.^(20)^

The present study has an important limitation of counting with two RCT that strictly answered the study question. However, this is an important indicator that more RCTs using different surgical techniques and energy delivery systems in association with tranexamic needed to assess its real-life effects on blood-loss-related outcomes and complications. As we saw during our search, the TURP procedure is well studied, whereas B-TURP and laser assisted nucleation of the prostate requires more research on strategies to minimize blood loss.

## CONCLUSION

Available studies lack agreement in their results regarding the effect of tranexamic acid on reducing blood loss in patients undergoing B-TURP for benign prostatic hyperplasia treatment. Therefore, further research is required in this specific area. Overall, this study emphasizes the need for additional research on the use of tranexamic acid to reduce blood loss during B-TURP.

## Data Availability

The data that support the findings of this study are available from the corresponding author, Cha JD, upon reasonable request.
